# Advanced diffusion imaging reveals microstructural characteristics of primary CNS lymphoma, allowing differentiation from glioblastoma

**DOI:** 10.1093/noajnl/vdae093

**Published:** 2024-06-08

**Authors:** Urs Würtemberger, Martin Diebold, Alexander Rau, Veysel Akgün, Lucas Becker, Jürgen Beck, Peter C Reinacher, Christian A Taschner, Marco Reisert, Luca Fehrenbacher, Daniel Erny, Florian Scherer, Marc Hohenhaus, Horst Urbach, Theo Demerath

**Affiliations:** Department of Neuroradiology, Medical Center—University of Freiburg, University of Freiburg, Freiburg, Germany; Institute of Neuropathology, Medical Center—University of Freiburg, University of Freiburg, Freiburg, Germany; IMM-PACT Clinician Scientist Program, University of Freiburg, Freiburg, Germany; Department of Neuroradiology, Medical Center—University of Freiburg, University of Freiburg, Freiburg, Germany; Department of Diagnostic and Interventional Radiology, Medical Center—University of Freiburg, University of Freiburg, Freiburg, Germany; Department of Neuroradiology, Medical Center—University of Freiburg, University of Freiburg, Freiburg, Germany; Department of Neuroradiology, Medical Center—University of Freiburg, University of Freiburg, Freiburg, Germany; Department of Neurosurgery, Medical Center—University of Freiburg, University of Freiburg, Freiburg, Germany; Fraunhofer Institute for Laser Technology, Aachen, Germany; Department of Stereotactic and Functional Neurosurgery, Medical Center—University of Freiburg, University of Freiburg, Freiburg, Germany; Department of Neuroradiology, Medical Center—University of Freiburg, University of Freiburg, Freiburg, Germany; Department of Stereotactic and Functional Neurosurgery, Medical Center—University of Freiburg, University of Freiburg, Freiburg, Germany; Department of Medical Physics, Medical Center—University of Freiburg, University of Freiburg, Freiburg, Germany; Institute of Neuropathology, Medical Center—University of Freiburg, University of Freiburg, Freiburg, Germany; Institute of Neuropathology, Medical Center—University of Freiburg, University of Freiburg, Freiburg, Germany; Department of Medicine I, Medical Center—University of Freiburg, University of Freiburg, Freiburg, Germany; Department of Neurosurgery, Medical Center—University of Freiburg, University of Freiburg, Freiburg, Germany; Department of Neuroradiology, Medical Center—University of Freiburg, University of Freiburg, Freiburg, Germany; Department of Neuroradiology, Medical Center—University of Freiburg, University of Freiburg, Freiburg, Germany

**Keywords:** diffusion microstructure imaging, DMI, DTI, NODDI, PCNSL

## Abstract

**Background:**

Primary CNS lymphoma (PCNSL) and glioblastoma (GBM) both represent frequent intracranial malignancies with differing clinical management. However, distinguishing PCNSL from GBM with conventional MRI can be challenging when atypical imaging features are present. We employed advanced dMRI for noninvasive characterization of the microstructure of PCNSL and differentiation from GBM as the most frequent primary brain malignancy.

**Methods:**

Multiple dMRI metrics including Diffusion Tensor Imaging, Neurite Orientation Dispersion and Density Imaging, and Diffusion Microstructure Imaging were extracted from the contrast-enhancing tumor component in 10 PCNSL and 10 age-matched GBM on 3T MRI. Imaging findings were correlated with cell density and axonal markers obtained from histopathology.

**Results:**

We found significantly increased intra-axonal volume fractions (V-intra and intracellular volume fraction) and microFA in PCNSL compared to GBM (all *P* < .001). In contrast, mean diffusivity (MD), axial diffusivity (aD), and microADC (all *P* < .001), and also free water fractions (V-CSF and V-ISO) were significantly lower in PCNSL (all *P* < .01). Receiver-operating characteristic analysis revealed high predictive values regarding the presence of a PCNSL for MD, aD, microADC, V-intra, ICVF, microFA, V-CSF, and V-ISO (area under the curve [AUC] in all >0.840, highest for MD and ICVF with an AUC of 0.960). Comparative histopathology between PCNSL and GBM revealed a significantly increased cell density in PCNSL and the presence of axonal remnants in a higher proportion of samples.

**Conclusions:**

Advanced diffusion imaging enables the characterization of the microstructure of PCNSL and reliably distinguishes PCNSL from GBM. Both imaging and histopathology revealed a relatively increased cell density and a preserved axonal microstructure in PCNSL.

**Key Points**

- PCNSL is compared to GBM, characterized by increased cell density and preserved axonal frameworks, which is traceable with advanced diffusion imaging and histopathology.
- In this pilot study, MRI DTI, NODDI, and DMI metrics permit a reliable differentiation of PCNSL from GBM. The results require repetition in a larger patient cohort.

**Importance of the Study**

To the best of our knowledge, this study is the first to noninvasively characterize the microstructure of PCNSL using advanced multicompartmental diffusion models based on NODDI or DMI. Beyond previous studies indicating increased cellularity in PCNSL, compared to GBM we found evidence of preserved axonal frameworks in PCNSL. Numerous DTI-, NODDI-, and DMI-based diffusion metrics also allow for a good differentiation of PCNSL from GBM, which is of high clinical relevance as these tumor entities differ substantially in their diagnostic and therapeutic approach. These metrics are also reconciled with histopathologically increased cellularity and increased axonal remnants in PCNSL.

In adults, primary CNS lymphoma (PCNSL) is the second most frequent primary intracranial malignant brain tumor following gliomas.^1^ The distinction between these entities is crucial, as clinical management differs considerably. PCNSL is usually treated with high-dose chemotherapy after histopathological confirmation by stereotactic biopsy^2^ without gross resection, whereas GBM is treated by tumor excision and adjuvant radiochemotherapy.^3^ In addition, high-dose steroid therapy prior to biopsy can lead to nondiagnostic histopathology and is therefore usually avoided in PCNSL.^2^ Although the majority of PCNSL exhibit characteristic imaging features on MRI, differentiation from GBM can be challenging as both entities may show “atypical” features such as absent central necrosis in GBM^4,5^ or presence of central necrosis in PCNSL,^5–8^ especially in immunocompromised patients.^6–8^

In contrast to imaging, pathology allows for the reliable differentiation of PCNSL and GBM.^9^ PCNSL can show infiltrative spread and central necrotic areas like GBM, but is characterized by high nucleus–plasma-ratio and pattern less lymphocytic cellularity.^9–11^

The increased cellularity in PCNSL is commonly reflected by alterations in conventional diffusion-weighted imaging, with a reduction of the apparent diffusion coefficient (ADC).^12–15^ Beyond this, advanced diffusion imaging allows for a more specific, noninvasive approximation of the brain’s microstructure.^16–19^ However, diffusion tensor imaging (DTI) revealed conflicting results based on the measurement of fractional anisotropy (FA) to differentiate PCNSL from GBM with some studies revealing lower FA and ADC in PCNSL,^12^ others confirming lower mean diffusivity (MD) with elevated FA in PCNSL.^20^ Likely, both the tumor-related cell density and the (partially preserved) axonal background microstructure play a role in the nondirectional diffusivity (measured with ADC/MD) within cerebral neoplasms. The combined analysis using both DTI and novel multicompartmental diffusion MRI approaches such as Neurite Orientation Dispersion and Density Imaging (NODDI)^16^ or Diffusion Microstructure Imaging (DMI)^17^ might overcome these constraints as they allow for an even more precise assessment of the microstructure. These novel diffusion techniques involve the estimation of the relative axonal cellular and extra-axonal/extracellular component (DMI V-intra, DMI V-extra, DMI V-CSF, NODDI intracellular volume fraction [ICVF], NODDI V-ISO, and NODDI-OD) as well as the calculation of microFA and microADC. DMI and NODDI have previously been used to differentiate between GBM and brain metastases based on contrast-enhancing tumor^21^ and peritumoral signal alterations^22,23^ with histopathologically traceable correlates. In a previous study in patients with temporal lobe epilepsy, DMI was validated using ultrastructural histopathology in a subsample with reduced temporopolar axonal density.^24^

We hypothesize that PCNSL and GBM differ within the proliferative tumor component in terms of the aforementioned diffusion metrics and that these can be correlated histopathologically. We, therefore, sought to investigate microstructural DTI, DMI, and NODDI metrics within the contrast-enhancing tumor components of PCNSL in correlation with histopathology. Moreover, we tested the diagnostic value by distinguishing PCNSL from GBM.

## Materials and Methods

### Patient and Imaging Characteristics

This retrospective study was approved by the local Institutional Review Board (EK:400/20). All procedures performed in studies involving human participants followed the ethical standards of the institutional and national research committee and the 1964 Helsinki Declaration and its later amendments. Informed written consent was waived by the local ethics committee due to the purely retrospective analysis.

Within 4 years (01/2018–04/2023), a total of 20 patients with newly diagnosed PCNSL (*n* = 10) and age-matched GBM (*n* = 10) were enrolled (see also Supplementary Figure 1). Ten PCNSL patients were matched 1:1 with GBM patients in terms of age (±2 years age difference). Patients with relevant small vessel disease (Fazekas > 1), concomitant vascular lesions eg vascular malformations), or imaging features of neurodegenerative disorders (eg Alzheimer’s disease, frontotemporal lobar degeneration, and cerebral amyloid angiopathy) were excluded. Similarly, previous tumor resections and brain biopsies, prior radiation therapy, and poor image quality led to study exclusion. In addition, for some of the patients not included in this study, surgical planning was carried out based on external MRI images, which is partly due to reduced examination capacities during the corona pandemic.

Imaging was conducted with 3 Tesla MRI scanners (MAGNETOM Prisma and Prisma FIT, Siemens Healthcare) using a 64-channel head and neck coil. Diffusion MRI sequences were acquired with the following parameters: axial orientation, 42 slices, voxel size 1.5 × 1.5 × 3 mm^3^, TR 2800 ms, TE 88 ms, bandwidth 1778 Hz, flip angle 90°, simultaneous multiband acceleration factor 2, GRAPPA factor 2, 58 diffusion-encoding gradient directions per shell with *b*-factors 1000, and 2000 s/mm^2^, and 15 nondiffusion weighted images (interleaved during diffusion-encoding directions); this resulted in a total of 131 images.; acquisition time was 6 min and 22 s. High-resolution isotropic T1w postcontrast sequences were acquired 4–5 min after i.v. injection of 0.1 mmol/kg gadoteridol (Gd) (ProHance^®^, Bracco Imaging) with 3-dimensional (3D) magnetization-prepared 180° radio-frequency pulses and a rapid gradient-echo (MP-RAGE) sequence (repetition time: 2500 ms; echo time: 2.82 ms; flip angle: 7°, TI = 1100 ms; GRAPPA factor 2; 1.0 mm isotropic voxels; 192 contiguous sagittal slices).

### Image Postprocessing

Data processing was performed on a local instance of the postprocessing platform NORA (www.nora-imaging.org; last accessed on December 15, 2023). T1w image datasets were automatically segmented into white matter, gray matter, and cerebrospinal fluid (CSF) with SPM12 (Wellcome Centre for Human Neuroimaging).

Preprocessing of diffusion MRI data included denoising,^25^ Gibbs-ringing artifacts-correction,^26^ and up sampling to the isotropic resolution of 1.5 mm³.^17^ DTI measures were obtained from *b* = 0 and 1000 s/mm^2^ images using a publicly available open-source toolbox (https://www.uniklinik-freiburg.de/mr-en/research-groups/diffperf/fibertools.html) using the ordinary log-linear fitting, calculating the fractional anisotropy (FA) and also mean (MD), axial (aD), and radial diffusivity (RD). While the FA describes the extent of directional (anisotropic) diffusion, the MD is a rotationally invariant measure of the mean diffusion in each direction of a voxel. The aD, which describes the mean diffusion coefficient of water molecules diffusing parallel to a tract, and the rD, which describes the magnitude of diffusivity perpendicular to a tract, contribute to MD. NODDI-derived ICVF (or intra-neurite fraction), free water fraction (ISO-VF) and orientation dispersion (OD) were calculated with the accelerated microstructure imaging via convex optimization (AMICO)-method, a regularized version of NODDI with faster processing times due to the linearization of fitting procedures.^27^ DMI-based microFA, microADC, intra-axonal volume fraction (V-intra), extra-axonal volume fraction (V-extra), and free water fraction (V-CSF) were estimated using a Bayesian approach.^17^ MicroFA and microADC both are independent of the orientational distribution at the meso-scale, ie they do not depend on the relative orientation of the microstructural features within a voxel but depend on intrinsic microstructural features, which are in the range of several micrometers (defined by the diffusion-time/length of dMRI measurement).

**Figure 1.**
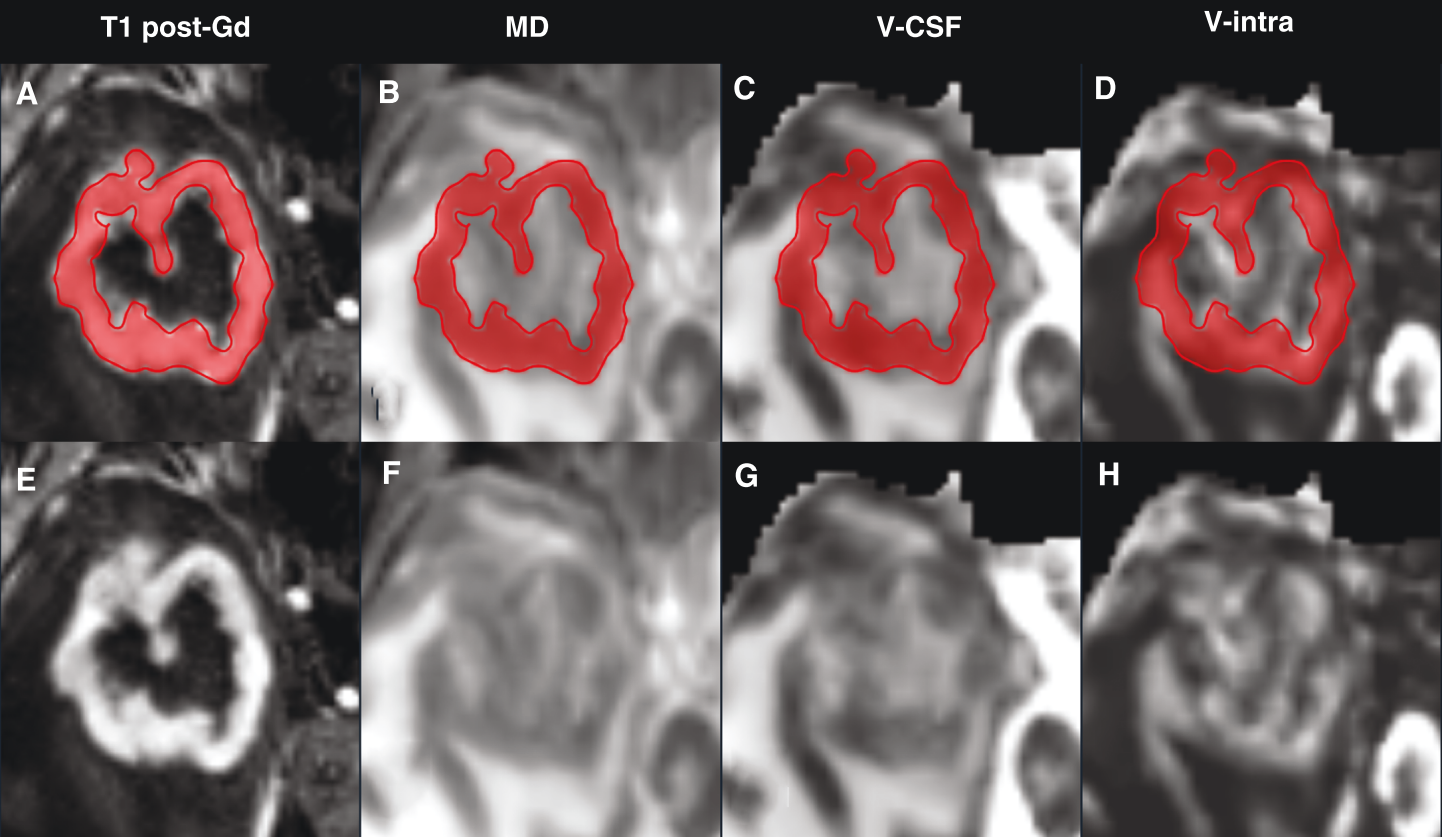
Presurgical (3T) MR imaging in a patient with an atypical (central necrotic) right temporal PCNSL. Representative axial images are shown in the upper row both with (A–D) and without (E–G) the corresponding ROI of the contrast-enhancing tumor component based on (A). Gd indicates Gadoteridol.

Whole tumor contrast-enhancing tumor components were manually segmented as regions of interest (ROI) by 1 neuroradiologist (5 years of clinical neuroimaging experience) and cross-checked by another neuroradiologist (7 years of clinical neuroimaging experience) by consensus on 3D T1w post-Gd datasets. Image data sets were first reviewed for motion artifacts. Then the regular coregistrations of the MPRAGE and diffusion data sets were checked. To account for potential partial volume effects, we carefully excluded noncontrast enhancing tumor margins, as illustrated in Figure 1.

### Histopathology

Histological analysis of contrast-enhancing tumor samples was performed according to standardized protocols of the local Institute of Neuropathology. In cases with GBM, the samples were analyzed from contrast-enhancing tumor parts in close temporal relation to the analyzed MR images; in cases with PCNSL, the tissue was obtained according to protocol utilizing stereotactic biopsies (except for 1 case in which GBM was suspected based on the initial MRI). A total of 20 samples (10 PCNSL/10 GBM) were processed by established diagnostic procedures for fixation in 4% paraformaldehyde, paraffin embedding, staining, and immunohistochemistry. Here, representative regions were assessed in Bielschowsky silver staining for the presence of axonal remnants and hematoxylin–eosin-staining (H&E) for cellular density. Due to scarce tissue from stereotactic biopsies, analysis was based on 3 (H&E) and 5 (Bielschowsky) randomly recorded high-power field images (400-fold magnification). The presence of axonal remnants was assessed using a binary code, while cell density was quantified using QuPath application^28^ (Version 0.3.2 with Java 16.0.2) on triplicate images. To limit analysis to tissue, ROI were identified through an artificial neural network algorithm pixel classifier trained for cells and extracellular matrix. Watershed-based cell detection was applied using standardized parameters (pixel size 2 μm, radius 14 μm, and sigma 5). Images were acquired using an Olympus BX40 microscope (Olympus K.K., Shinjuku) and a Leica DFC450 camera (Leica Microsystems) (for Bielschowsky) and on a Keyence BZ-X810 compact microscope (Keyence Corporation, for H&E). Figure 4 shows examples of PCNSL and GBM with anatomic and parametric MRI maps and histopathologic imaging.

### Statistical Analysis

The assumption for normal data distribution was tested with the Shapiro–Wilk test. Patients’ ages and histological outcomes were compared between PCNSL and GBM using the Mann–Whitney-*U* test. Sex was compared with the Chi-square test. ANOVA was conducted between ROI-derived diffusion metrics comparing PCNSL and GBM groups and pooled normal appearing white matter (NAWM) and Tukey post hoc Test was employed to account for multiple comparisons. Linear regression modeling with Spearman’s rank correlation coefficient was used to relate DTI-, NODDI-, and DMI-derived diffusion metrics to cellular density. The receiver-operating characteristic (ROC) curves of PCNSL and GBM MD, aD, microFA, V-intra, V-CSF, ICVF, V-ISO, and microADC were plotted. An α-level of 0.05 was considered statistically significant. All statistical analyses were performed using GraphPad Prism (version 9.3.1).

## Results

### Study Population

We report on 20 patients with contrast-enhancing intracranial mass lesions that underwent presurgical MRI including multishell dMRI. Of those, histopathology confirmed an IDH wild-type GBM in 10 patients (5 female; median age: 72.8; interquartile range (IQR) 68.4–78.0 years) whereas 10 patients (4 female; median age 72.5; IQR 68.4–80.3 years) had PCNSL. Ten PCNSL patients were matched 1:1 with GBM patients in terms of age (±2 years age difference). Both groups did not differ in terms of age (*P* = .91), sex (*P* = .65) or total volume of contrast-enhancing tumor components with a median volume in GBM of 12.45 ml [IQR 4.6–20.2 mL] and in PCNSL of 13.5 mL [IQR 10.5–17.2 mL] (*P* = .97).

### Diffusion Metrics in Contrast-Enhancing Areas of PCNSL and GBM

There was a significant overall group difference in MD [*F*(2,37) = 16.8, *P* < .001], rD [*F*(2,37) = 8.31, *P* = .001], aD [*F*(2,37) = 8.68, *P* < .001], FA [*F*(2,37) = 16.0, *P* < .001], microFA [*F*(2,37) = 17.5, *P* < .001], V-intra [*F*(2,37) = 12.6, *P* < .001], V-CSF [*F*(2,37) = 12.1, *P* < .001], ICVF [*F*(2,37) = 18.7, *P* < .001], V-ISO [*F*(2,37) = 7.88, *P* = .001] and OD [*F*(2,37) = 11.9, *P* < .001]. There was no significant difference on the group level regarding V-extra [*F*(2,37) = 0.008, *P* = .99]. Individual group values are presented in Supplementary Table 1.

**Figure 2.**
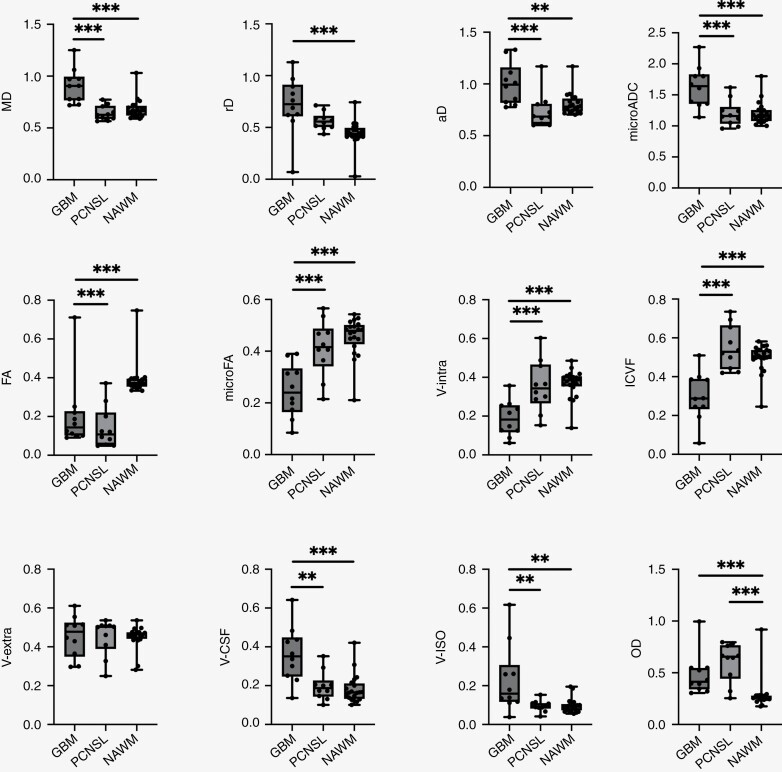
DTI, DMI, and NODDI metrics in contrast-enhancing tumor areas in 10 patients with GBM and 10 patients with PCNSL. Compared to GBM, PCNSL showed a significant shift toward lower MD, aD, and also microADC, lower free water fractions reflected by lower V-CSF and V-ISO, relatively increased microADC, microFA, V-intra, and ICVF, whereas no significant between-group differences were found regarding FA, V-extra, and OD. GBM = glioblastoma; PCNSL = primary CNS lymphoma; NAWM = normal appearing white matter; **P < .01; ***P < .001.

Tukey’s post hoc tests revealed there was a significant increase in MD in GBM compared to both PCNSL (*P* < .001, 95% CI = 1.26, 3.37) and NAWM (*P* < .001, 95% CI = 1.07, 2.89), a significant increase in rD in GBM compared to NAWM (*P* < .001, 95% CI = 0.70, 2.44), a significant increase in aD in GBM compared to both lymphomas (*P* = .001, 95% CI = 0.76, 2.76) and also NAWM (*P* = .005, 95% CI = 0.47, 2.16). Regarding the microADC, we noted a significant increase in GBM compared to both PCNSL (*P* < .001, 95% CI = 0.82, 2.82) and NAWM (*P* < .001, 95% CI = 0.91, 2.70). There was a significant reduction in FA in both GBM (*P* = .001, 95% CI = –2.35, –0.63) and lymphomas (*P* < .001, 95% CI = –2.92, –1.09) compared to NAWM, but a significant relative decrease in microFA in GBM compared to both PCNSL (*P* < .001, 95% CI = –2.81, –0.80) and NAWM (*P* < .001, 95% CI = –3.22, –1.32). Regarding multicompartmental DMI metrics, we noted a significant reduction in DMI V-intra in GBM compared to both PCNSL (*P* = .001, 95% CI = -2.73,-0.74) and NAWM (*P* < .001, 95% CI = -2.77,-0.97), a relative increase in V-CSF in GBM compared to both PCNSL (p = 0.002, 95% CI = 0.70,2.68) and also NAWM (*P* < .001, 95% CI = 0.94,2.73), which was accompanied by a significant reduction in NODDI-ICVF in GBM compared to both PCNSL (*P* < .001, 95% CI = -3.63,-1.46) and also NAWM (*P* < .001, 95% CI = –2.90, –1.07). We also noted a relative increase in V-ISO in GBM compared to both PCNSL (*P* = .006, 95% CI = 0.50, 2.44) and also NAWM (*P* = .002, 95% CI = 0.59, 2.29), and compared to NAWM a significant increase of OD in both GBM (*P* = 0.018, 95% CI = 0.29, 1.94) and PCNSL (*P* < .001, 95% CI = 0.92, 2.70). The distribution of values is presented in Figure 2, exemplary cases are presented in Figure 4.

### ROC Analysis

**Figure 3.**
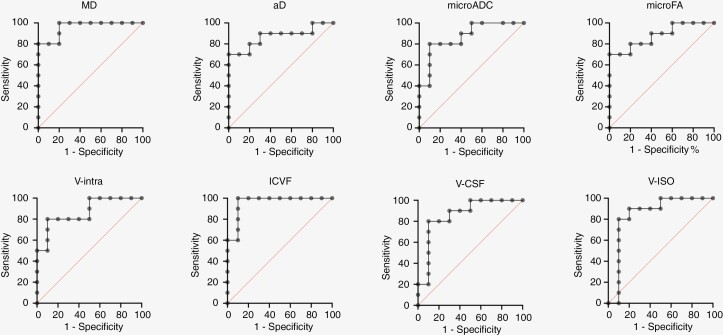
ROC curves of 10 patients with GBM and 10 with PCNSL illustrating high predictive values regarding the presence of a PCNSL, for mean and axial diffusivity (MD and aD), DMI-derived microscopic apparent diffusion coefficient (microADC), microscopic fractional anisotropy (microFA), DMI-derived intra-axonal, and also CSF volume fractions (V-intra and V-CSF), NODDI-derived intracellular and isotropic volume fractions (ICVF, V-ISO; AUC in all >0.840, highest for MD and ICVF, both with an AUC of 0.960).

Building on the systematic differences regarding MD, aD, microFA, V-intra, V-CSF, ICVF, V-ISO, and microADC values within contrast-enhancing tumor components between PCNSL and GBM, we conducted a ROC analysis defining these parameters as dependent variables and PCNSL and GBM groups as class variables. This model supported the affiliation to “PCNSL” with lower MD values (area under the curve [AUC] = 0.960; 95% CI, 0.88–1.00; *P* < .001), lower aD (AUC = 0.870; 95% CI, 0.70–1.00; *P* < .01), higher microFA (AUC = 0.880; 95% CI, 0.73–1.00; *P* < .01), higher V-intra (AUC = 0.870; 95% CI, 0.71–1.00; *P* < .01), lower V-CSF (AUC = 0.860; 95% CI, 0.69–1.00; *P* < .01), higher ICVF (AUC = 0.960; 95% CI, 0.87–1.00; *P* < .001), lower V-ISO (AUC = 0.850; 95% CI, 0.65–1.00; *P* < .01), and lower microADC (AUC = 0.870; 95% CI, 0.71–1.00; *P* < .01). ROC curves are presented in Figure 3.

**Figure 4.**
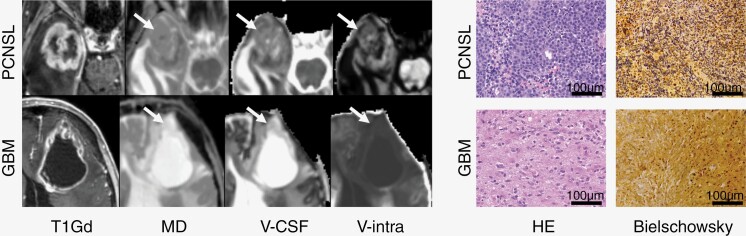
Left column: Axial T1-post-Gd images and parametric maps for mean diffusivity (MD), free water (V-CSF), and intra-axonal volume fraction in a case of an atypical (central necrotic) right temporal PCNSL (upper row, same case as presented in Figure 1) and a left frontal GBM (lower row). In PCNSL, increased cell density was accompanied by an increased MD and lowered free water fraction (V-CSF) accompanied by a relatively increased intra-axonal volume fraction (V-intra) within the contrast-enhancing tumor component. Right column: Representative Hematoxylin Eosin (HE) and Bielschowsky’s silver staining for nerve fibers in cases with a PCNSL and GBM (recorded at 400-fold magnification). The scale bar corresponds to 100 μm. Cell density was significantly higher in PCNSL compared to GBM. Axonal remnants were found in a significantly higher proportion of PCNSL. Both dMRI and histopathology indicated a relative preservation of axonal frameworks in PCNSL.

A model equally weighted for sensitivity and specificity considerably improved the detection of PCNSL (sensitivity, 100%; specificity 80%) for MD when applying the estimated optimal cutpoint of <0.776. There also was a substantial diagnostic value (sensitivity, 100%; specificity 90%) detectable for ICVF with a cutpoint of >0.409. The associated confidence intervals, as well as the sensitivity and specificity of the other analyzed metrics (aD, microADC, microFA, V-intra, V-CSF, and V-ISO), are presented in Supplementary Table 2.

### Histopathology

**Figure 5.**
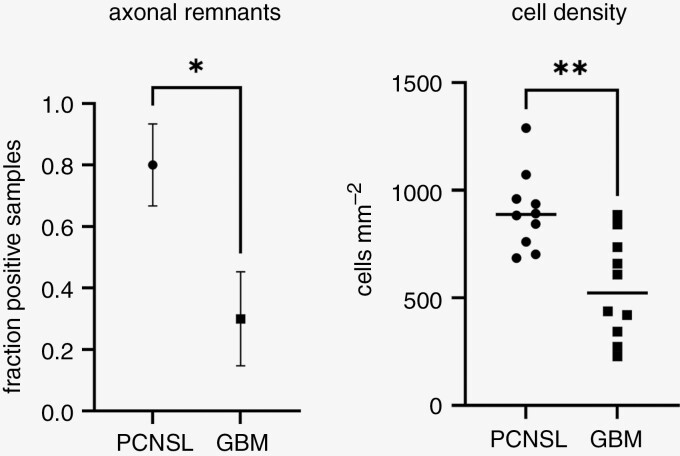
Axonal remnants were found in a significantly higher proportion of PCNSL (*P* = .02). Cell density was significantly higher in PCNSL compared to GBM (*P* = .001).

Analysis of neuronal structures revealed a significantly higher prevalence of axonal remnants within PCNSL (8/10 cases) than in GBM (3/10 cases), in 5 randomly selected high-power field images (Welch’s *t*-test, *P* = .02). PCNSL also exhibited increased cell density (mean = 902/mm²) compared to GBM (mean = 543/mm²) in vital tumor areas (Welch’s *t*-test, *P* = .001). Exemplary cases including parametric diffusion maps are presented in Figures 4 and 5.

### Correlation Analysis

Results of a simple linear regression test revealed a moderate positive association between average cellular density and ICVF (*R*² 0.32, *P* = .009) and a moderate negative association between cellular density and MD (*R*² 0.25, *P* = .03). No other DTI, DMI, or NODDI metrics revealed statistically significant associations (positive trends were observed for microFA (*R*² 0.07), and V-intra (*R*² 0.07), and negative trends for V-ISO (*R*² 0.19) and FA (*R*² 0.7)).

**Figure 6.**
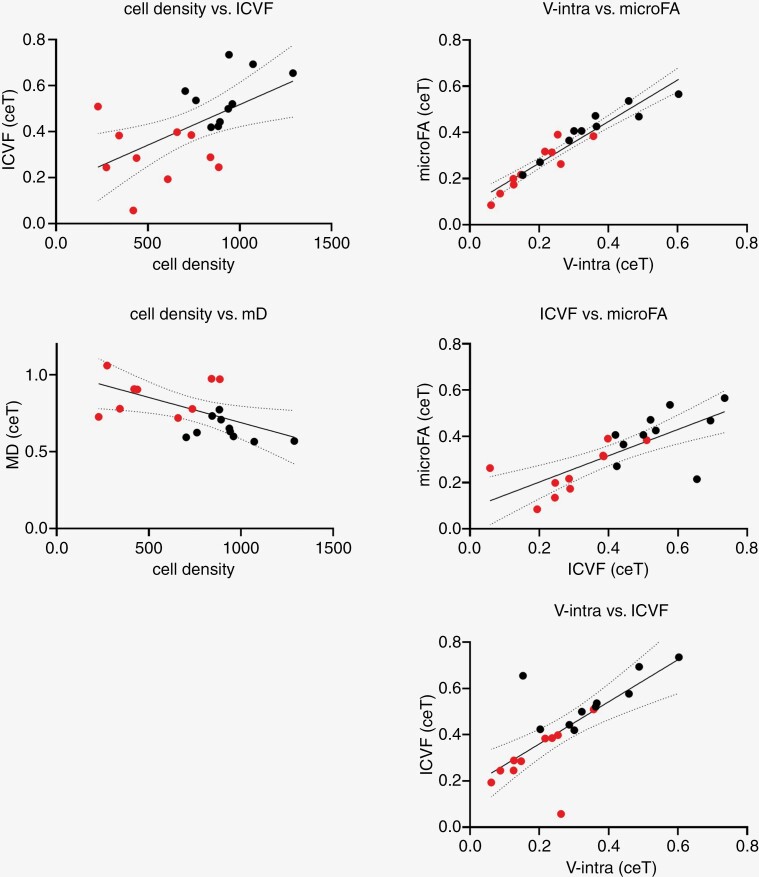
Linear regression revealed a moderate positive association between average cellular density and ICVF (*R*² 0.32, *P* = .009) and a moderate negative association between cellular density and MD (*R*² 0.25, *P* = 0.03) and the stringent association between ICVF, microFA, and V-intra. Plots are presented with individual values of PCNSL in red and GBM in black and corresponding 95% CI.

Strong positive associations between V-intra and microFA (*R*² 0.91, *P* < .001), ICVF and microFA (*R*² 0.54, *P* < .001), and V-intra and ICVF (*R*² 0.55, *P* < .001) were found (Figure 6).

## Discussion

In our pilot study, we were able to elucidate the distinct tumor microstructure in PCNSL by using a combination of diffusion MR imaging metrics involving DTI and DMI/NODDI, which was consistent with histologically increased cell density and relative preservation of axonal frameworks in PCNSL. Both MD and ICVF appear to be particularly suitable as discriminative parameters to differentiate between PCNSL and GBM, which is of particular clinical relevance.

A fundamental assumption leading to the broad application of diffusion sequences in neuro-oncology studies, such as in PCNSL and GBM,^4,12,14,15,20,29^ is that densely packed tumor cells with a high nuclear to cytoplasmic ratio leads to a restriction in the free diffusion of water molecules, which translates into reduced ADC and MD. Deviating from this, individual studies also report a lack of correlation between the mean ADC and nuclei count in PCNSL ^30^) and even a positive correlation between MD and cellularity in gliomas^31^. However, the majority of studies, as reported in a large meta-analysis^32^ , describe a negative correlation between cellularity and ADC. Our finding of reduced MD is largely confirmed by the available literature, with 1 study demonstrating (histologically) increased cellularity in PCNSL accompanied by a significantly reduced ADC,^14^ and others confirming decreased ADC and MD in PCNSL.^12,20^ In our sample, we also found a significant reduction of aD in PCNSL and a trend towards reduced rD, largely corresponding to a measurable reduction of not only nondirectional but also directional diffusivity metrics in a cell-rich environment. In PCNSL, we measured decreased microADC consistent with increased cellularity, although not significantly correlating with cell density in histopathology. Whereas ADC only allows for a coarse approximation of the cellular density within PCNSL, microADC is largely independent of effects related to the orientation distribution of the axons. Thus, we anticipate a better approximation of cell density measurements in PCNSL, which needs to be confirmed by experimental studies. In summary, we have strong evidence that histologically increased cell density is measurable in PCNSL by using both DTI and more recent DMI metrics.

Regarding axonal remnants within central tumor components, we found relative preservation of axonal frameworks in PCNSL in histopathology using Bielschowsky staining. No significant differences in FA between PCNSL and GBM were observed, however, both were significantly decreased compared to NAWM. While FA has been reported as a sensitive parameter to microstructural alterations such as demyelination,^33,34^ its utility in regions of complex white matter structure has been questioned particularly in brain areas with increased OD.^35^ Interestingly, compared to GBM we measured similar FA-values but a significant increase in microFA in PCNSL, approximating values within NAWM. This finding may correlate with a preserved anisotropy at the microscopic level, which is only detectable in PCNSL. Further evidence of preserved axonal remnants in PCNSL was provided by, compared to GBM, significantly increased NODDI-ICVF as well as DMI- and V-intra, which we primarily interpret as relative preservation of axonal microstructure in PCNSL and pronounced loss of axons in GBM. The potential suitability of multicompartment diffusion methods for estimating axonal density is supported by several preliminary studies. For example, reduced axonal density was detected in patients with temporopolar gray-white matter blurring in hippocampal sclerosis with correlating electron microscopy,^24^ as were NODDI-based axonal loss and damage in amyotrophic lateral sclerosis^36^ or multiple sclerosis.^37^ The robust positive correlations between ICVF, V-intra, and microFA in this study highlight the association between axonal density and microstructural anisotropy. Reduced axonal density in GBM compared to PCNSL may either be related to microenvironmental alterations^38^ or direct mechanical stress caused by the tumor, which can lead to neuronal loss,^39^ as well as white matter demyelination.^40^ Axonal preservation may also explain why therapeutic response in PCNSL, in our view, commonly is not accompanied by significant parenchymal defects. To the best of our knowledge, our study is the first in which axonal density in PCNSL was evaluated jointly based on histopathology and MRI. The extent to which the axonal structures are functionally impaired and truly preserved in the long term should be the aim of future studies including clinical and longitudinal imaging data.

Regarding free water imaging, we detected decreased V-CSF as well as V-ISO in PCNSL. These findings were accompanied by a decrease in MD as well as reduced rD in PCNSL. In summary, this most likely reflects decreased free water in the interstitial space with lower nondirectional diffusivity due to higher cell density and preservation of axons in PCNSL.^41^

We found no significant differences in OD between PCNSL and GBM, with both groups having significantly increased values compared to NAWM. This can be well explained by the increased degree of axonal dispersion with reduced directional diffusivity prevailing in PCNSL, and axonal degradation, predominant in GBM. Well-compatible with this, we also observed an inverse relationship of OD to FA, which has previously been described experimentally and also been applied in model tumors.^16,21^

In the future, in addition to the imaging method presented here, further non or minimally invasive techniques may provide an alternative to stereotactic brain biopsy or accelerate the postbiopsy diagnostic workup, such as the detection of PCNSL tumor DNA using liquid biopsy^42,43^ or use of flow cytometry, with significantly faster results compared to classic immunohistochemistry.^44^ On the one hand, these methods would have the advantage of being less invasive due to the absence of a stereotactic puncture; on the other hand, the latter methods would make it possible to obtain genetic material, which is certainly an advantage over MRI-based procedures. However, for ICVF and MD in particular, we were also able to demonstrate substantial diagnostic value with regard to PCNSL detection with an excellent sensitivity of 100% and high specificities of 80% (MD) and 90% (ICVF) in our small cohort, which is, regarding MD/ADC, conformable with results from previous studies.^12^

Besides the obvious intrinsic limitations of a retrospective study, our study is limited by the rather small patient population, also prohibiting the evaluation of within-group differences between typical and atypical (necrotic) PCNSL. Even though corrections were made for multiple tests, the results, also including calculations of sensitivity and specificity, should be interpreted with caution. Besides that, it should be noted that the multicompartmental model assumptions of DMI and NODDI were not primarily designed for tumor microenvironments and therefore need to be further investigated, which may promote optimization of more specific diffusion models.^45,46^ Nevertheless, larger studies are needed here to verify the results.

## Conclusion

Multicompartment diffusion imaging indicates increased cell density and preserved axonal frameworks in PCNSL, which is histopathologically traceable. Both MD and ICVF appear to be particularly suitable as discriminative parameters to differentiate between PCNSL and GBM, which is of particular clinical relevance.

## Supplementary material

Supplementary material is available online at *Neuro-OncologyAdvances* (https://academic.oup.com/noa).

vdae093_suppl_Supplementary_Figure_S1

vdae093_suppl_Supplementary_Table_S1

vdae093_suppl_Supplementary_Table_S2

## Data Availability

The anonymized data presented in this study are available on reasonable request from the corresponding author (U.W.).
