# Supplementary figures and images for: Advanced diffusion imaging reveals microstructural characteristics of primary CNS lymphoma, allowing differentiation from glioblastoma
Source: Neurooncol Adv. 2024 Jun 8;6(1):vdae093. doi: 10.1093/noajnl/vdae093 (PMC11214103; doi:10.1093/noajnl/vdae093)

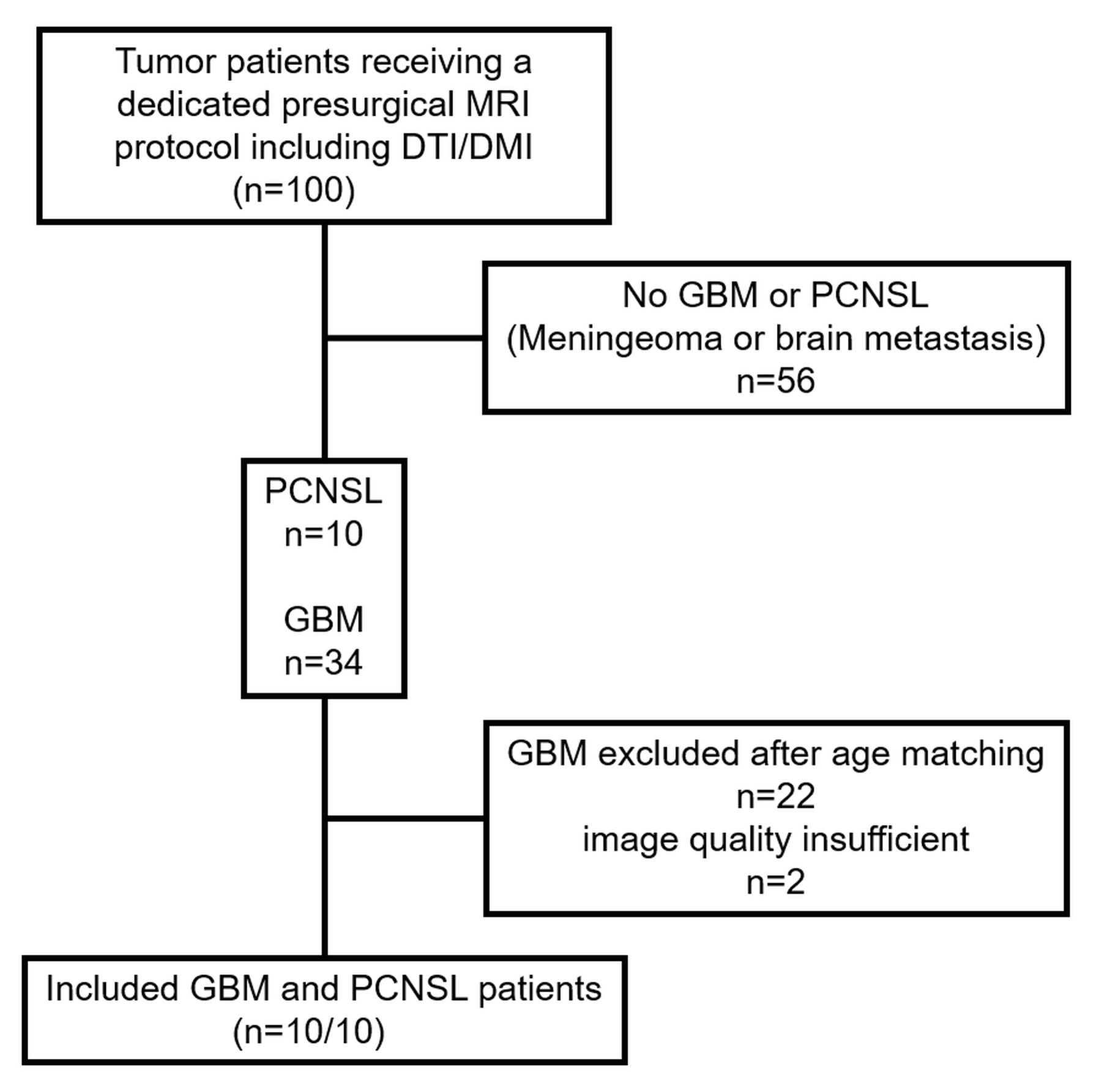

Supplement: vdae093_suppl_Supplementary_Figure_S1 [file vdae093_suppl_supplementary_figure_s1.jpeg]
