# Supplementary material for: Advanced diffusion imaging reveals microstructural characteristics of primary CNS lymphoma, allowing differentiation from glioblastoma
Source: Neurooncol Adv. 2024 Jun 8;6(1):vdae093. doi: 10.1093/noajnl/vdae093 (PMC11214103; doi:10.1093/noajnl/vdae093)
Supplement: vdae093_suppl_Supplementary_Table_S1 [file vdae093_suppl_supplementary_table_s1.docx]

|  | **Group** | **MD** | **rD** | **aD** | **FA** | **microFA** | **V_intra** | **V_extra** | **V_CSF** | **ICVF** | **ISOVF** | **OD** | **microADC** |
| --- | --- | --- | --- | --- | --- | --- | --- | --- | --- | --- | --- | --- | --- |
| Mean | GBM | 0.907 | 0.715 | 1.000 | 0.206 | 0.248 | 0.188 | 0.454 | 0.361 | 0.300 | 0.230 | 0.481 | 1.630 |
|  | PCNSL | 0.645 | 0.563 | 0.738 | 0.143 | 0.413 | 0.355 | 0.452 | 0.198 | 0.550 | 0.095 | 0.602 | 1.200 |
|  | NAWM | 0.682 | 0.444 | 0.806 | 0.390 | 0.456 | 0.368 | 0.450 | 0.184 | 0.496 | 0.098 | 0.286 | 1.210 |
| Median | GBM | 0.905 | 0.724 | 0.993 | 0.144 | 0.240 | 0.182 | 0.478 | 0.351 | 0.287 | 0.159 | 0.413 | 1.640 |
|  | PCNSL | 0.627 | 0.556 | 0.684 | 0.108 | 0.416 | 0.343 | 0.503 | 0.187 | 0.528 | 0.091 | 0.655 | 1.160 |
|  | NAWM | 0.661 | 0.439 | 0.780 | 0.373 | 0.479 | 0.385 | 0.464 | 0.165 | 0.506 | 0.092 | 0.259 | 1.160 |
| SD | GBM | 0.167 | 0.286 | 0.201 | 0.186 | 0.103 | 0.092 | 0.106 | 0.144 | 0.127 | 0.177 | 0.202 | 0.336 |
|  | PCNSL | 0.071 | 0.084 | 0.170 | 0.109 | 0.109 | 0.134 | 0.094 | 0.075 | 0.113 | 0.030 | 0.189 | 0.209 |
|  | NAWM | 0.097 | 0.126 | 0.106 | 0.087 | 0.075 | 0.073 | 0.059 | 0.075 | 0.073 | 0.036 | 0.152 | 0.184 |
| Minimum | GBM | 0.719 | 0.070 | 0.776 | 0.091 | 0.085 | 0.062 | 0.297 | 0.136 | 0.058 | 0.039 | 0.304 | 1.140 |
|  | PCNSL | 0.566 | 0.436 | 0.602 | 0.049 | 0.215 | 0.153 | 0.250 | 0.101 | 0.420 | 0.042 | 0.254 | 0.957 |
|  | NAWM | 0.590 | 0.028 | 0.703 | 0.333 | 0.211 | 0.139 | 0.282 | 0.101 | 0.246 | 0.056 | 0.176 | 1.000 |
| Maximum | GBM | 1.250 | 1.130 | 1.330 | 0.711 | 0.390 | 0.357 | 0.611 | 0.642 | 0.510 | 0.617 | 0.996 | 2.270 |
|  | PCNSL | 0.773 | 0.713 | 1.170 | 0.372 | 0.566 | 0.603 | 0.536 | 0.352 | 0.735 | 0.153 | 0.795 | 1.620 |
|  | NAWM | 1.030 | 0.743 | 1.170 | 0.747 | 0.543 | 0.486 | 0.536 | 0.421 | 0.582 | 0.195 | 0.918 | 1.800 |
