# Supplementary material for: Advanced diffusion imaging reveals microstructural characteristics of primary CNS lymphoma, allowing differentiation from glioblastoma
Source: Neurooncol Adv. 2024 Jun 8;6(1):vdae093. doi: 10.1093/noajnl/vdae093 (PMC11214103; doi:10.1093/noajnl/vdae093)
Supplement: vdae093_suppl_Supplementary_Table_S2 [file vdae093_suppl_supplementary_table_s2.docx]

|  | **Cutoff Value** | **Sensitivity %** | **95% CI (%)** | **Specificity %** | **95% CI (%)** |
| --- | --- | --- | --- | --- | --- |
| MD | < 0.776 | 100 | 72.25-100 | 80 | 49.02-96.45 |
| aD | < 0.814 | 80 | 49.02-96.45 | 80 | 49.02-96.45 |
| microADC | < 1.295 | 80 | 49.02-96.45 | 90 | 59.58-99.49 |
| microFA | > 0.341 | 80 | 49.02-96.45 | 80 | 49.02-96.45 |
| V-intra | > 0.275 | 80 | 49.02-96.45 | 90 | 49.02-96.45 |
| ICVF | > 0.409 | 100 | 72.25-100 | 90 | 59.58-99.49 |
| V-CSF | < 0.218 | 80 | 49.02-96.45 | 90 | 59.58-99.49 |
| V-ISO | < 0.117 | 90 | 59.58-99.49 | 80 | 49.02-96.45 |
